# Free, healthy school lunches in New Zealand: A Value for Investment analysis

**DOI:** 10.1186/s12889-025-24529-8

**Published:** 2025-10-21

**Authors:** Carolina Mejía Toro, Julian King, Sally Mackay, David Tipene-Leach, Boyd Swinburn

**Affiliations:** 1 School of Population Health, University of Auckland, Grafton, Auckland, 1023 New Zealand; 2 Julian King & Associates Limited, PO Box 44-111, Point Chevalier 1246, Auckland, New Zealand; 3 The University of Melbourne, Melbourne, Australia; 4 Te Kura I Awarua Rangahau Māori Research Centre, Eastern Institute of Technology, Hawke’s Bay, New Zealand, 501 Gloucester Street, Taradale, Napier 4112 New Zealand

**Keywords:** School meals, Food environments, Policy, Universality, Value for investment, Program evaluation

## Abstract

**Background:**

Universal, healthy school meal programs can address poor nutrition, improve educational outcomes and job and life opportunities, and support environmental sustainability. While many countries have extensive experience with such programs, New Zealand's initiative (Ka Ora, Ka Ako) was launched in 2020 as part of the COVID-19 recovery strategy and, by 2024, reached over 236,000 (~ 27%) school students.

**Objectives:**

This study aimed to assess the value generated from the investment (~ NZD320 million/year) in Ka Ora, Ka Ako and its potential for further value creation.

**Methods:**

The study employed the participatory Value for Investment framework to assess the program across five economic domains: effectiveness, efficiency, economy, equity, and cost-effectiveness. Four workshops with key community, government, and research stakeholders co-developed 21 evaluation criteria and rated the program’s performance based on evidence from program monitoring data, national research and international studies.

**Results:**

Ka Ora, Ka Ako rated very well overall, with seven criteria rated as excellent, 12 as good, two as adequate, and none as poor. All six primary outcomes set as the original purpose of the program, relating to alleviating hunger, improving nutrition, improving health and wellbeing, reducing financial burdens on families, reducing barriers to education and supporting local economies, were rated good (4) or excellent (2). The program's continuity was rated only as adequate due to announced major budget cuts and uncertainty about future funding. Although enhancing environmental sustainability was not part of the program’s original purpose, some sustainability criteria—packaging and food waste management—were rated as good. However, the criterion for sustainable food procurement systems was rated only as adequate. In light of these findings, the study assessed Ka Ora, Ka Ako as providing very good value for investment overall.

**Conclusions:**

The use of the Value for Investment approach was key because school lunch programs have many dimensions and the participatory research processes of the approach enabled stakeholders to emphasize the program’s wide-ranging social and health benefits while identifying areas for improvement. While Ka Ora, Ka Ako rated very well across most of the 21 value dimensions, there are major concerns that this level of quality will not be upheld under the program’s budget cuts from 2025 onwards.

**Supplementary Information:**

The online version contains supplementary material available at 10.1186/s12889-025-24529-8.

## Introduction

### The problem of child hunger, food insecurity and malnutrition in all its forms

A compelling body of evidence demonstrates that hunger and malnutrition, especially in childhood, significantly increase the risk of illness, particularly from noncommunicable diseases (NCDs). These conditions can also cause irreversible damage to physical growth, cognitive development, and psychological wellbeing, with effects that can span generations [1–4]. As a result, food-insecure children face serious disadvantages that limit their opportunities and outcomes in health, education, employment, and income [3, 5]. These disadvantages lead to substantial losses in human and economic capital, including increased healthcare and welfare costs [1, 6, 7].

Hunger is defined internationally as the uncomfortable or painful physical sensation caused by insufficient dietary energy consumption [8]. Malnutrition, on the other hand, results from deficiencies, excesses, or imbalances in the intake or absorption of energy and nutrients [9]. Both hunger and malnutrition can arise from food insecurity—that is, inadequate access to safe, nutritious, and sufficient food—as well as from unhealthy environments or poor childcare practices [8, 9]. These challenges are not limited to low- and middle-income countries, as household food insecurity is significant and increasing in many high-income nations [10]. Simultaneously, malnutrition in all its forms—including undernutrition, micronutrient deficiencies, overweight, and obesity—is the leading cause of disability-adjusted life years lost in every country [11].

New Zealand portrays the case of a high-income country [12, 13] with a very high prevalence of child food insecurity (27%) [14] and overweight and obesity (31.5%) [15], with marked disparities by ethnicity and level of socio-economic deprivation [14, 15].

### School meal programs

A widely adopted strategy for enhancing food security is the provision of free meals in schools, a practice implemented across numerous countries for over a century. Originally intended to alleviate child poverty, school meal programs have, since the second half of the twentieth century, evolved to address malnutrition in all its forms and prevent its adverse effects on health and learning [16, 17]. These programs emphasize nutritional balance and food safety, providing a significant portion of students'daily dietary requirements [18–20]. Economic crises, such as the 2008 global downturn and the COVID-19 pandemic, have underscored the critical role of universal free school meal programs in reducing financial strain on households while improving health, educational outcomes, and local economic activity [16, 21–24].

Healthy, free school meals are a ‘triple-duty’ action, simultaneously helping to combat obesity, undernutrition, and climate change—the three most pressing challenges for global health in the twenty-first century [11, 17]. Consequently, international policy platforms, including the United Nations Food Systems Summit, advocate for their role in advancing population nutritional health and sustainable food systems [25–28].

### School lunches in New Zealand

In 2019, the New Zealand centre-left Labour government introduced a free school lunch program pilot, which, by January 2020, was covering about 21,000 (2.5%) of school students attending primary schools with high levels of socioeconomic disadvantage [29–31]. In response to the spike in food insecurity from the COVID-19 lockdowns, the scheme was rapidly expanded to cover all students in primary and secondary schools in the highest quartile of disadvantage, using COVID economic recovery funding. By May 2024, the program, called ‘Ka Ora, Ka Ako’ (‘being well means being able to learn’), reached more than 236,000 primary and secondary learners (over 27% of school learners) across 1,013 schools [31, 32], The stated purposes of the program were to help alleviate hunger and food insecurity, improve school students’ nutritional health and well-being, remove barriers to learning at school and support local employment [33, 34].

A new centre-right coalition government was formed in November 2023, and, over the course of this study, the Minister responsible for Ka Ora, Ka Ako, was seeking ways to cut the program costs, culminating in a May 2024 announcement that from 2025, the program budget would be reduced around NZD107 million, about one third from its initial budget (NZD323 million) [35, 36].

### Current evidence on Ka Ora, Ka Ako

From 2020 to 2023, multiple evaluations of Ka Ora, Ka Ako were undertaken along with a comprehensive review of both New Zealand and international evidence on the benefits of Ka Ora, Ka Ako and similar international programs [22, 37–42]. The New Zealand Ministry of Education commissioned three evaluations focusing on impacts such as hunger and nutrition, wellbeing, school attendance, procurement, and employment [43–46] and the implementation of lunch provision models developed by Māori organizations [46]. Additionally, the Ministry of Education had internal and public quantitative and qualitative monitoring data regarding lunch provider performance (including delivery and safety), user and provider satisfaction, teacher engagement, food waste, food surplus, and the nutritional quality and environmental footprint of the lunches [32, 45–49]. Other independent evaluations of Ka Ora, Ka Ako included qualitative interviews with principals, families, and students and quantitative measurements of student eating patterns and anthropometry [21, 22, 50, 51].

Thus, multiple sources of data were available on the program, but they had not been collectively analyzed for a return-on-investment analysis. Cost–benefit analyses require the benefits to be monetized, and cost-effectiveness analyses require the benefits to be consolidated into a common metric (e.g., quality-adjusted life-years), but neither of these approaches could comprehensively assess broader advantages of healthy school meal programs—such as well-being, mental health, and community cohesion—which are difficult to monetize or quantify. One return-on-investment method, Value for Investment (VfI), allows for a more comprehensive analysis of resource use and consequential returns. It integrates theoretical and practical approaches from evaluation and economics, accommodating mixed methods and stakeholder participation to assess complex policies and programs [52–54].

### Aims

This VfI study aimed to comprehensively assess the value generated from the investment in Ka Ora, Ka Ako and use the evaluation results to assess how well resources have been used in the program, its potential to deliver additional value to stakeholders, and identify key areas for learning that could inform the future development or enhancement of free school meal policies in New Zealand and internationally.

## Methods

### Value for investment

An *evaluation-specific approach* [55] was applied. This approach defines evaluation as the systematic determination of merit, worth or significance [55] and sets out to provide robust information about *how good* an intervention is, whether it is *good enough*, and how it can be improved [56]. Evaluation is “a judgment-oriented practice – it does not aim simply to describe some state of affairs but to offer a considered and reasoned judgment about the value of that state of affairs” [57]. This characteristic of evaluation, which sets it apart from research, is underpinned by a logic that involves synthesizing empirical evidence through the lens of explicit criteria (aspects of performance) and standards (levels of performance) to transparently judge value [55, 58] – a process known as *evaluative reasoning*. In this context, *value* is understood as the regard or importance assigned to something. Consistent with this interpretation, interventions do not possess inherent value; rather, people determine their worth by assigning value based on their perspectives and experiences [54].

The Value for Investment (VfI) evaluation system was selected as the framework to evaluate Ka Ora, Ka Ako because it applies an evaluation-specific approach to assess value-for-money – that is, how *well resources* are used to produce the expected value, whether *enough* value is created, and how more value can be produced [53, 59, 60]. While VfI has been applied in social development and public health contexts, this is the first time it is used for evaluating universal school food programs.

The VfI framework is grounded in four principles: interdisciplinarity, mixed methods, evaluative reasoning, and participation. Interdisciplinarity merges insights from evaluation and economics, while mixed methods integrate quantitative and qualitative data for a more comprehensive understanding – for example, through triangulation to identify areas of agreement or disagreement between different evidence sources. Evaluative reasoning provides the means to make warranted judgements from evidence, making judgements transparent, traceable, and challengeable. Lastly, the participatory approach ensures stakeholders have a voice in the evaluation process, from evaluation co-development to validation of findings. This engagement enhances the relevance and usability of evaluations by incorporating the values of those involved in the program's design and delivery, rather than solely those of the evaluation team [53, 54].

**Fig. 1:**
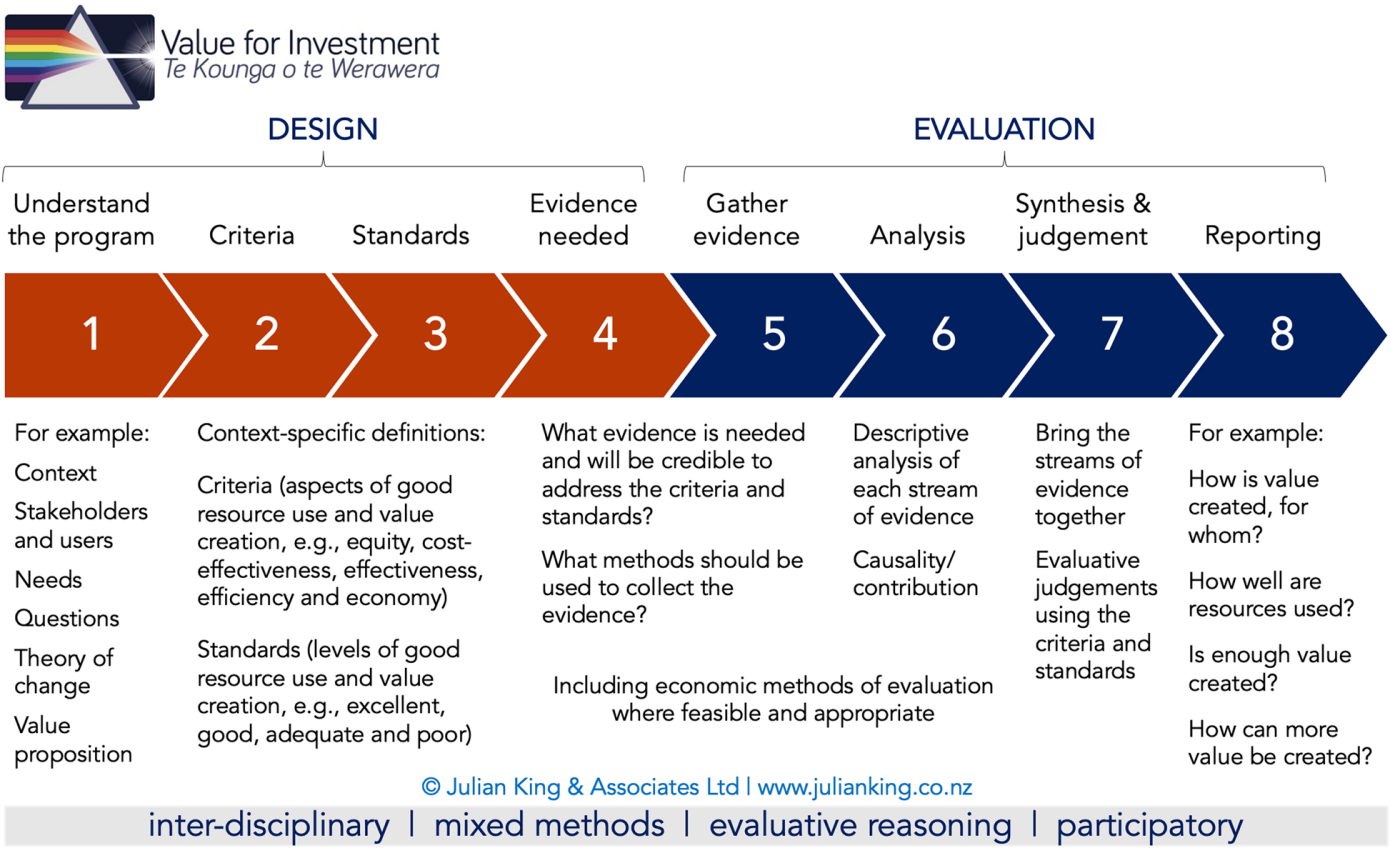
The Eight Steps of Value for Investment Evaluation. Source: Julian King & Associates. What is the Value for Investment? In: Value for Investment Resources, by Julian King & Associates Ltd. 2024. Image reproduced with the permission of its author and rights holder

Figure 1 below provides an overview of the eight steps within the VfI evaluation system and their definitions.

### Design

Ka Ora, Ka Ako was officially introduced in New Zealand in January 2020, and this research was conducted between November 2023 and August 2024. Ethics approval for the research was obtained from the Eastern Institute of Technology (EIT) Research Ethics Committee.

To address steps 1–3 of the VfI evaluation design, two workshops were held with community and government Ka Ora, Ka Ako stakeholders to gather input on the values they expected the program to deliver, who would benefit, and the overall value proposition. These inputs were then shaped into the VfI ‘Value List’, which guided the evaluation team in developing a framework comprising 21 evaluation sub-criteria, organized into five economic domains known as the 5-E Framework: Effectiveness, Efficiency, Equity, and Cost-effectiveness [61]. This evaluation framework enabled a transparent, evidence-based assessment of Ka Ora, Ka Ako’s performance against the program’s expected values. The criteria were reviewed and validated by stakeholders during a third workshop to create the final, validated set of criteria. For VfI steps 4–6, the evaluation team identified, collated and analyzed evidence from previous Ka Ora, Ka Ako impact evaluations and monitoring data, as well as national and international research to create an evidence base for the assessment of each criterion. Sources of evidence were identified through consultations with a diverse range of key stakeholders, including community representatives, school principals, program suppliers, independent researchers, program evaluators, and local and national government representatives. Several stakeholders recommended the analysis of specific research publications, independent and government reports, previous government and independent evaluations, and key websites for review.

In addition, government actors involved in program monitoring provided relevant data on implementation design and operational metrics, including procurement prices, resource and waste management, periodic surveys with school staff and suppliers, and routine quality audits.

For VfI step 7, a fourth virtual workshop with key stakeholders—including representatives from schools, the community, relevant government agencies, and researchers involved in the Ka Ora, Ka Ako evaluation. Participants were provided with the summary of evidence for each criterion, and then, individually rated the level of program performance (*Excellent, Good, Adequate, Poor*) for each of the 21 criteria on a virtual poll.

Table 1 below shows the standards used and their generic definitions for rating performance [60]. Each criterion typically had multiple evidence elements to consider, so stakeholders had to integrate the evidence as a whole and rate the performance according to the generic definitions.

**Table 1:** Value for investment standards for program and policy evaluation

Standard	Generic definition
*Excellent*	Meeting or exceeding all reasonable expectations/targets, bearing in mind the context. Room for incremental improvements
*Good*	Generally meeting reasonable expectations/targets, allowing for minor exceptions. Some improvements needed
*Adequate*	Not meeting expectations/targets but fulfilling minimum requirements and showing acceptable progress overall. Significant improvements needed
*Poor*	Not fulfilling minimum requirements or not showing acceptable progress overall. Urgent improvements needed

Ahead of the rating workshops with stakeholders (step 7), there was a need to disseminate preliminary findings to inform governmental budget decisions on the future of the program’s funding. Thus, in March 2024, the research team that gathered and collated the evidence prepared provisional ratings for the evaluation criteria, which were published in a blog as preliminary results [62] (Supplementary Material 1). Next, the virtual workshop for stakeholder ratings took place in May 2024, and the final results were disseminated to the participants.

### Stakeholder recruitment and procedures

This VfI study was part of the ‘Nourishing Hawke’s Bay (NHB): He wairua tō te kai (the spirit in the food)’ program of research on child nutrition and food security in the Hawke’s Bay region of New Zealand. Community stakeholders were purposively recruited from schools, community organizations and individuals involved in Ka Ora, Ka Ako using the NHB networks from previous studies [21, 22, 50, 51, 63] and snowball techniques. Government stakeholders were officials from the Ministry of Education and Ministry of Health involved in Ka Ora, Ka Ako. The research stakeholders who were involved in the final rating workshop were those in the NHB team who were not directly involved in running the VfI study or co-authors of this paper.

The workshops were structured as follows:

- Workshop 1: Community insights, construction of the value proposition and evaluation criteria

This workshop was face-to-face with Hawke’s Bay community stakeholders in November 2023 and covered steps 1-3 (Fig. 1). Following discussions about the context of Ka Ora, Ka Ako and this VfI study, participants brainstormed in small groups the expected impacts from the program, the value of those impacts to different stakeholder groups, and the mechanisms through which Ka Ora, Ka Ako converts resources invested into social value. A draft summary Value Proposition statement (VP) was developed. In the VfI system, the VP statement is contextually defined and collectively built through discussions and agreements among stakeholders [53]. After the workshop, the expected values were grouped into broad categories guided by the thematic analysis methods proposed by Gajaweera and Johnson [64] The criteria were placed into the domains of the 5-E framework along with sub-criteria reflecting additional aspects of good resource use [60]. The 5-E domains are: *effectiveness* (achieving expected values/outcomes), *economy* (good stewardship of resources to acquire inputs), *efficiency* (using inputs productively to deliver outputs), *equity* (reducing inequities and promoting fairness through program design, delivery, and outcomes), and *cost-effectiveness* (maximizing value/outcomes for the resources invested) [53, 60]

- *Workshop 2: Government insights, additions to the value proposition and evaluation criteria*

This workshop was held face-to-face with government officials in February 2024 to build on steps 1-3. Participants brainstormed potential outcomes from Ka Ora, Ka Ako and contributed to refining the expected values and the draft VP that emerged from Workshop 1. The research team then merged insights from both workshops.

- *Workshop 3: Validation of the value propositions*

**Fig. 2:**
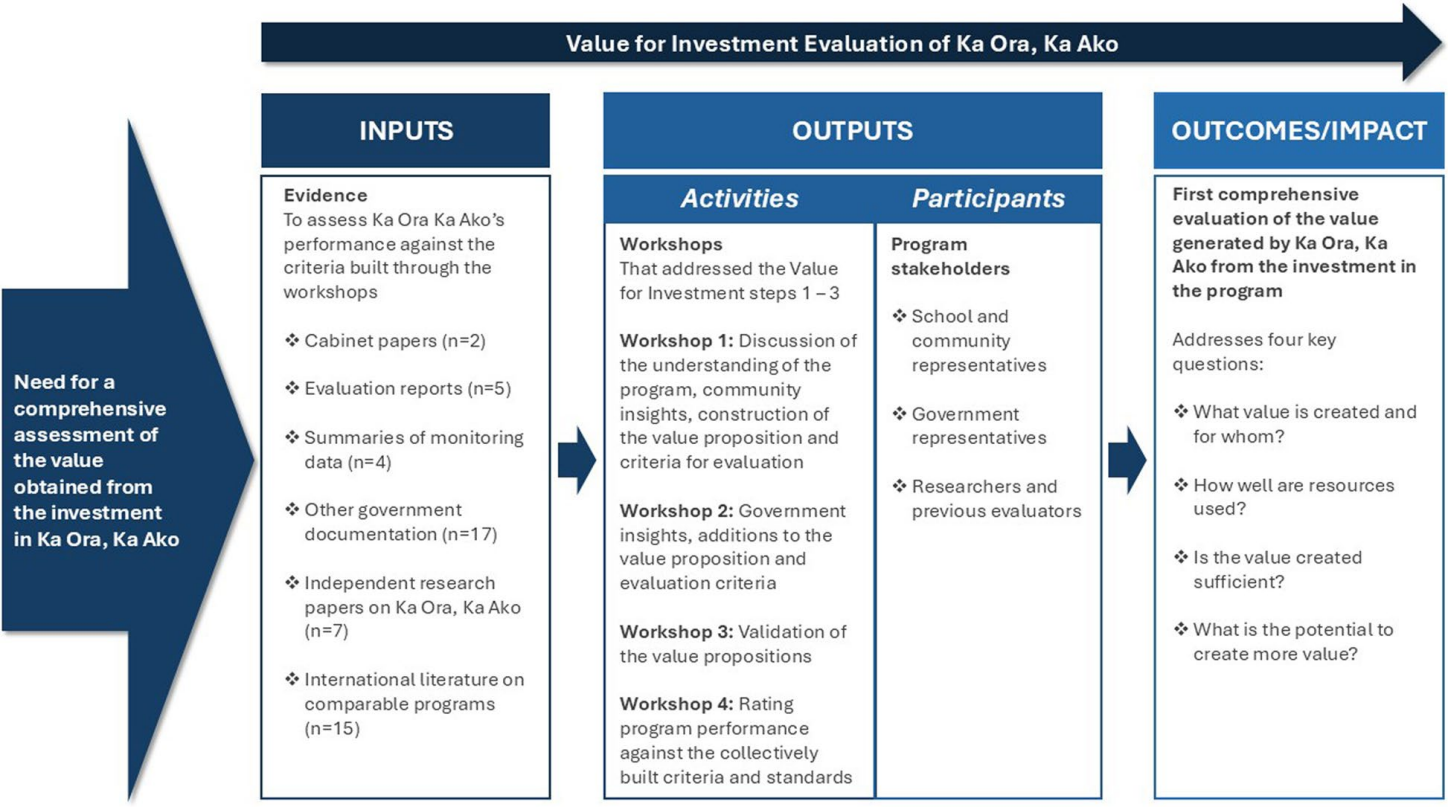
Logic model of inputs and outputs for the evaluation process of Ka Ora, Ka Ako

In March 2024, stakeholder groups from Workshops 1 and 2 gathered online to review and validate the merged values and the draft VP. Based on their feedback, a final VP, Value List, and table of evaluation criteria were developed (see Table 2 and Fig. 2 in results).

**Table 2:** The Value List for Ka Ora, Ka Ako

*Improved health and wellbeing*	*Improved learning outcomes*	*Improved local economies and community cohesion*	*Improved environmental sustainability*
Through: ·Alleviating hunger at school ·Improving eating patterns of students and whānau ·Improving access to healthy foods ·Helping students to attain healthy growth ·Improved diet-related health outcomes (dental health, NCD risks) ·Improving mental health ·Improving mana and self-esteem ·Improving cultural identity by: (i) embedding Mātauranga Māori (traditional knowledge of the Maori people in New Zealand) , (ii) preventing poverty stigma	Through: ·Alleviating hunger at school and improving students’ and whānau (family) food security ·Improving students’ nutritional status ·Targeting schools most in need ·Reducing the stigma of poverty (through universality design) ·Embedding manaakitanga (hospitality and generosity, showing kindness and respect for others) and environmentally sensitive nutrition education in the curriculum ·Increasing educational attainment and classroom engagement ·Improving attendance through removing food insecurity-related barriers and improving motivation ·Increasing high-school retention	Through: ·Investing in distribution infrastructure and economically viable systems to include local food in lunches ·Enacting procurement policies to support sustainable and local content of school lunches and including Iwi procurement	Through: ·Embedding sustainability considerations in meal planning ·Embedding sustainability in procurement and contract policies ·Reducing packaging and food waste (including collaboration with food rescue organizations) ·Linking to education about sustainability ·Including more locally produced foods ·Supporting more small-scale producers

- *Workshop 4: Rating of the evidence*

Following the collation of evidence (VfI steps 5-6), this virtual workshop was held in May 2024 to present the evidence. Participants used this evidence to rate the program's performance against the 5-E domains and 21 sub-criteria framework using the VfI generic standards outlined in Table 1. Attendees included community and government stakeholders from Workshops 1 and 2, as well as NHB researchers involved in Ka Ora and Ka Ako research but who were not conducting the VfI study. The polling feature of the virtual communication platform Zoom® was utilized to gather ratings from individual stakeholders. Each VfI standard (Table 1) was scored as follows: Excellent = 4, Good = 3, Adequate = 2, Poor = 1. The average scores from all stakeholders were then converted into a final rating per criterion, using the following ranges: Excellent (3.5-4.0), Good (3.0-3.49), Adequate (1.5-2.99), and Poor (1.0-1.49).

### Evidence collection, analysis, and assessment

Multiple sources of secondary evidence were used to collate the evidence base for each criterion. These included: cabinet papers (*n* = 2), Ka Ora, Ka Ako evaluation reports (*n* = 5), summaries of internal and public monitoring data of the Ministry of Education (*n* = 4), independent research on Ka Ora, Ka Ako (*n* = 7), other government documentation (*n* = 17), and international literature on similar school lunch programs where no New Zealand data was available (*n* = 15).

Specifically, the evaluation reports focused on the impacts on students’ hunger and nutrition, wellbeing, and school attendance, as well as employment and procurement, including the implementation of models developed by Māori organizations [14, 18–20]. Monitoring data encompassed quantitative and qualitative information gathered through termly surveys with schools and providers, in-situ observations, and complaints received through official complaint channels. This data covered lunch provider performance (including delivery, organoleptic and safety compliance), user and provider satisfaction, teacher engagement, food waste and surplus management, and the nutritional quality of the lunches [10, 15, 19, 20, 24, 25]. Independent research on Ka Ora, Ka Ako included measurements and analyses of student eating patterns and anthropometry [13, 21–23], as well as qualitative interviews with principals, families, and students. Moreover, the international literature reviewed focused on cost–benefit analyses and the long-term (> 5 years) impact of similar school lunch programs on the health, wellbeing, educational outcomes, school retention, and future income of the students.

All relevant information was entered into a matrix in Microsoft Excel, where key details for each VfI evaluation criterion were extracted, collated, and summarized. This summarized information was then compiled into a PowerPoint slide deck and sent to participants of Workshop 4 in advance. During the workshop, each criterion was explained one at a time, allowing participants to ask questions before providing their ratings.

Figure 2 presents a logic model that outlines the key inputs and outputs involved in the evidence collection, analysis, and assessment process for the VfI evaluation of Ka Ora, Ka Ako.

## Results

### Context, criteria and standards (Steps 1–3)

Workshop 1 included 43 participants: 7 representatives from local schools and community organizations, 1 representative from a charity; 30 representatives from local food provision entities, and 5 regional representatives from the Ministry of Education and the Ministry of Social Development. Small group brainstorming sessions generated numerous potential values for inclusion in the Value List and summary VP, which were then refined in a plenary session. The research team further developed these concepts into a draft Values List, organizing them into four umbrella themes: Improved Health and Wellbeing, Improved Learning Outcomes, Improved Local Economies and Community Cohesion, and Improved Environmental Sustainability. Thereafter, a summary draft VP was created.

Workshop 2 included national-level government officials from the Ministry of Education (*n* = 6) and the Ministry of Health (*n* = 5). They brainstormed the potential values of Ka Ora, Ka Ako and then reviewed the draft findings from Workshop 1. There was a considerable overlap in expected values between participants of Workshops 1 and 2.

**Fig. 3:**
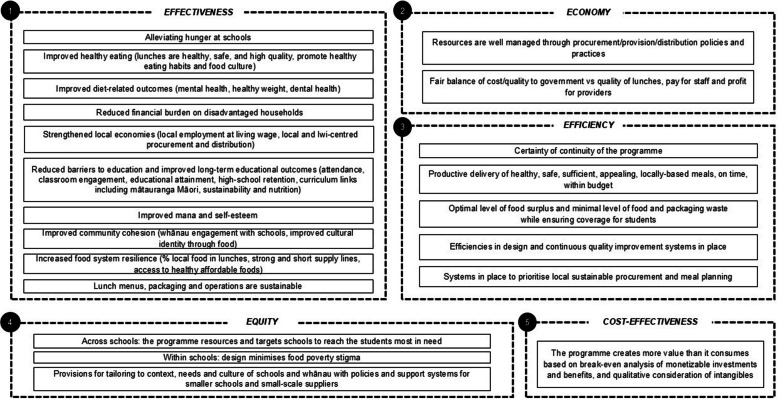
Final criteria used in the Value for Investment analysis, organized according to the 5-E Framework

After Workshop 2, the research team further refined the concepts in the draft Value List, clarified their translation into a total of 21 evaluation criteria grouped for insertion into the 5-E framework, and improved the wording of the draft VP. This grouping did not result in a loss of values since, within one criterion, there were often several lines of evidence which could capture the nuanced differences in values with a grouping into one criterion. Changes were reviewed and validated with the participants from Workshops 1 and 2 in a third combined virtual workshop. The final Value List (Table 2) turned into program evaluation criteria grouped in the 5-E framework (see Fig. 3).

The final Value Proposition featured a comprehensive list of attributes and outcomes reflecting the multi-dimensional nature of Ka Ora, Ka Ako: “A healthy, tasty, sustainable, equitable, free school lunch system that contributes to improving food security, nutritional health and wellbeing, learning outcomes, environmental sustainability, and local economies and communities.”

The final set of criteria shown in the 5-E Framework (Fig. 3) formed the structure of the evaluative judgements. Note the predominance of effectiveness criteria, which were foremost in the stakeholders’ minds and the added economic criteria to fill out the 5-E framework. The criterion ‘Fair balance of cost/quality to government vs quality of lunches, pay for staff and profit for providers’ was included under the Economy domain rather than the Efficiency domain. This was because the government procured the lunches for schools based on a range of criteria and not only based on who had the lowest price. In purchasing decisions, the Ministry of Education purposely strove to achieve a fair balance between getting a low price, maintaining good quality lunches, and ensuring that business margins were fair to ensure economic sustainability and efficiency. Similarly, the criterion ‘Optimizing levels of food surplus and minimizing waste while ensuring coverage for students’ recognized that there is an optimal level of average food surplus (uneaten lunches) which is not zero, since that would jeopardize full coverage of lunches for students. Since it was known that there had been no formal cost-effectiveness studies undertaken on Ka Ora, Ka Ako, that criterion was framed as at least a break-even position of monetized costs and benefits.

### Evidence evaluation (Steps 4–6)

Forty-four evidence sources (24 from the Ministry of Education, 20 from independent national and international sources) were used to address the 21 evaluation criteria. No longitudinal analyses on the impact of Ka Ora, Ka Ako on learning and educational attainment outcomes and no cost–benefit or formal economic analyses of the program had been done. International evidence of similar programs was used to fill these gaps.

The full evidence base for the 21 criteria was summarized into a slide deck (one slide per criterion) (Supplementary Material 2– slide deck as PDF). The format of each slide was designed to be concise enough for quick review during the rating workshop, while still providing sufficient detail for researchers to elaborate on and reference the underlying evidence.

### Stakeholder rating of program performance (Step 7)

The final one-hour workshop was conducted virtually and attended by 7 school and community stakeholders, 4 government officials and 8 researchers. The slide deck had been sent to participants beforehand, and each slide with the evidence for each of the 21 criteria was explained to participants in the workshop. They had an opportunity to ask questions before they individually rated each criterion according to the generic standards (Table 1). Table 3 shows the results from the Zoom poll from the 19 stakeholders who attended Workshop 4 and joined the poll to rate the 21 criteria to evaluate Ka Ora, Ka Ako.

**Table 3:** Stakeholder votes and overall ratings of Ka Ora, Ka Ako’s performance

	Criterion	Excellent (n)	Good (n)	Adequate (n)	Poor (n)	Average score		Final rating
*Effectiveness*								
1	*Alleviating hunger at schools	9	9	1	-	3.4	GOOD	
2	*Improved healthy eating	12	7	-	-	3.6	EXCELLENT	
3	*Improved diet-related outcomes	8	10	1	-	3.4	GOOD	
4	*Reduced financial burden on disadvantaged households	14	5	-	-	3.7	EXCELLENT	
5	*Strengthened local economies	9	9	1	-	3.4	GOOD	
6	*Reduced barriers to education and improved long-term educational outcomes	9	9	1	-	3.4	GOOD	
7	Improved mana and self-esteem	8	10	1	-	3.4	GOOD	
8	Improved community cohesion	6	8	5	-	3.1	GOOD	
9	Increased food system resilience	7	4	8	-	2.9	GOOD	
10	Lunch menus, packaging and operations are sustainable	5	7	4	3	2.7	GOOD	
*Economy*								
11	Resources are well managed through policies and practices	4	7	8	-	2.8	GOOD	
12	Fair balance of cost/quality to government vs quality of lunches, pay for staff and profit for providers	15	4	-	-	3.8	EXCELLENT	
*Efficiency*								
13	Certainty of continuity of the program	1	4	5	9	1.8	ADEQUATE	
14	Productive delivery of healthy, safe, sufficient, appealing, locally-based meals, on time, within budget	14	4	1	-	3.7	EXCELLENT	
15	Optimal level of food surplus and minimal level of food and packaging waste while ensuring coverage for students	9	6	3	1	3.2	GOOD	
16	Efficiencies in design and continuous quality improvement systems in place	13	4	2	-	3.6	EXCELLENT	
17	Systems in place to prioritise local sustainable procurement and meal planning	1	7	7	4	2.3	ADEQUATE	
*Equity*								
18	Across schools: program resources and targets schools to reach students most in need	4	4	10	1	2.6	GOOD	
19	Within schools: design minimises food poverty stigma	19						
20	Provisions for tailoring to context, needs and culture of school and whānau with policies and support systems for smaller schools and small-scale suppliers	12	5	2	-	3.5	EXCELLENT	
*Cost-effectiveness*								
21	The program creates more value than it consumes	16	1	2	-	3.7	EXCELLENT	

Asterisk (*): Criteria that correspond to the original aims of the program as defined in the Cabinet papers

Overall, the program rated very well, with seven criteria rated as excellent, 12 as good, two as adequate, and none as poor. Notably, five of the 12 scores rated as good were just below the cut-off for excellent, with scores of 3.4. There was unanimous agreement that criterion 19—regarding the ability of the design of the programme to minimize food poverty stigma—was excellent. This is attributable to the universal provision of lunches that ensures students with food insecurity are not singled out. While most criteria showed good consensus, three exhibited a broad distribution of ratings: criterion 10 (sustainability of lunch menus, packaging, and operations), criterion 13 (certainty of program continuity), and criterion 17 (prioritization of local sustainable procurement and meal planning).

In the preliminary ratings conducted by the research team prior to the fourth workshop, 12 criteria were rated as excellent, 4 as good, 4 as adequate, and 1 as poor (see Appendix 1). Criterion 13,"Certainty about Program Continuity,"was rated poor by the researchers because the assessment took place in March 2024, before the New Zealand Government's 2024 Budget was finalized. At that time, the program’s funding beyond 2024 was uncertain. However, following the budget announcement, Ka Ora, Ka Ako was allocated two additional years of funding, though at a significantly reduced investment level. Additionally, criterion 21 (cost-effectiveness) was rated adequate by the research team but excellent by workshop participants. The evaluators regarded the adequate rating as a conservative estimate due to the lack of New Zealand data on monetizable benefits, relying instead on international research from similar programs.

## Discussion

### Interpretation of the findings

This study used the VfI system to assess the value of New Zealand’s free school lunch program, Ka Ora, Ka Ako, which is currently delivered to about a quarter of school learners through a universal provision in schools in the areas of greatest socio-economic deprivation. The process of determining the expected values from the program’s value proposition and then rating the program’s performance against the evidence was highly participatory, with input from community and government stakeholders. Overall, the program was considered very good value for investment, with 19 out of 21 evaluation criteria being rated as either good or excellent. These ratings are more than a mere opinion poll because they reflect an explicit interpretation of the evidence through the lens of agreed-upon criteria and standards. This process mitigates against individual subjectivity, strengthening inter-rater reliability and validity of the conclusions reached [53, 54, 60].

Participants identified a wide array of values, which we consolidated into 21 criteria for assessment. This aligns with national and international evidence highlighting the extensive benefits of free lunch programs, impacting students, families, schools, communities, and food systems [22, 38, 40–42, 65]. The values defined by participants extended beyond the original goals of the program as defined by the government [33, 34], particularly in terms of its contribution to improving environmental sustainability. While there was a lack of formal cost-effectiveness analyses on Ka Ora, Ka Ako, and insufficient data on food and packaging waste since the program's inception, the evidence base upon which assessments were made ranged from good to strong. In addition, the generally high level of agreement among participants regarding the rating of program performance further enhances the robustness of the overall scorecard. This high level of monitoring and evaluation is not typical for government programs.

We had to use international evidence for the cost-effectiveness of similar programs to Ka Ora, Ka Ako. For example, a review of formal economic analyses of school meals in comparable high-income countries found that investment in universal school meals delivers a return of 2.5 times to 7 times in health and economic benefits [38, 40, 65].

The broader distribution of ratings for the two criteria related to environmental sustainability (10 and 17) likely reflected the varying levels of emphasis placed on this aspect across different schools. While environmental sustainability concerning packaging and food waste was not included in Ka Ora, Ka Ako’s original objectives, efforts to develop policies, monitoring systems, key performance indicators for suppliers, and enhanced technical support and guidelines for schools and suppliers were implemented from 2024 [48, 49].

The criterion related to the certainty of program continuity (criterion 13) received mixed ratings. At the outset of this study, Ka Ora, Ka Ako’s funding was only secure through the end of 2024, as it was part of the COVID-19 economic recovery package [66]. However, prior to the final workshop, where stakeholders rated the program’s performance, the New Zealand government announced an extension of the program for an additional two years, albeit with a significant reduction in funding per lunch per child [35]. As a result, the news of continued funding, albeit at a lower level, was met with mixed reactions.

In contrast, there was unanimous agreement on the high value of universal lunch provision within participating schools, highlighting its crucial role in preventing food poverty stigma. This aligns with international evidence supporting the effectiveness of universal approaches in reducing stigma associated with poverty [16, 41, 42, 67].

### Implications of the findings

The Ka Ora, Ka Ako program represents the largest intervention aimed at improving child nutrition in New Zealand's history. Despite its brief implementation period, the program has demonstrated strong performance across a wide range of outcomes.

The findings of this study indicate that stakeholders have high expectations for the program across multiple domains, which is appropriate given the significant investment of taxpayers’ money. Stakeholders expressed strong support for the program's original objectives, all of which rated well: alleviating hunger in schools, improving child nutrition and health, financially supporting disadvantaged households, strengthening local economies, and reducing barriers to educational engagement and attainment.

However, stakeholders closely connected to the program also recognize its broader potential benefits. These include advancing educational and cultural goals—such as incorporating mātauranga Māori (traditional Māori knowledge) into the curriculum—and contributing to environmental sustainability efforts.

### Implications for New Zealand’s food policy context

The World Health Organization (WHO) and expert reports provide evidence-based recommendations and technical advice on the highest impact and best value policies to implement to help curb diet-related NCDs [28, 68, 69]. New Zealand has adopted very few of these recommended food policies, falling behind international best practices in areas such as mandatory healthy school food policies [70], taxing sugary drinks and unhealthy foods [71, 72], banning unhealthy food marketing to children [73, 74], and establishing clear front-of-pack warning labels [75, 76]. This context underscores the importance of retaining and expanding the Ka Ora, Ka Ako program.

About half of the children living in moderately food insecure homes and a third of those living in severely food insecure homes are not attending schools covered by Ka Ora, Ka Ako [77]. If New Zealand enacted policies to reduce its unacceptably high poverty rates [78], this would deal with the root cause of high food insecurity. In the meantime, free school lunches for all schools can substantially help to alleviate some of the most detrimental consequences of child food poverty, which include low educational attainment, poor health, reduced self-esteem, and anti-social behaviours [22, 37, 38, 41, 42, 50, 79]. This is why advocacy groups have called for expanding Ka Ora, Ka Ako, growing its purpose to include sustainability, and making it a core part of government operating budgets rather than having it compete for new funding year-on-year [80].

The budget cuts to the program for 2025–6 were made in the name of efficiency [81]. However, from the stakeholders’ perspective, the greatest threat to efficiency arises from the uncertainty surrounding future funding for the program. This uncertainty creates significant barriers for caterers, suppliers, schools, and local councils to invest in the necessary infrastructure—such as premises, equipment, systems, staff training, and processes—that would maximize efficiencies, reduce waste, and achieve the broader goals of the Ka Ora, Ka Ako program. Moreover, stakeholders viewed the budget cuts as a threat to several key outcomes, including the nutritional quality of the lunches, the economic benefits to local communities through employment, the potential loss of local food networks, and the risk of undermining the principle of ‘universality’.

### Improving nutrition and food security

The persistence of food and nutrition issues, such as food insecurity, stunting, and obesity, has prompted many countries to implement or enhance school feeding programs [16, 19, 28, 38, 40]. These efforts have intensified in response to the acute negative impacts of the COVID-19 pandemic on food security [24, 82, 83] alongside the support garnered from the 2021 United Nations Food Systems Summit [24, 26, 84]. In New Zealand, childhood obesity and household food insecurity, which were already high pre-pandemic [77], spiked over the Covid lockdowns [85], making programs like Ka Ora, Ka Ako critical investments to address both immediate and long-term challenges. The VfI analysis presented in this study provides compelling justification for this policy response.

Moreover, findings from this study align with existing research indicating that school food programs are generally very cost-effective [38, 40, 65]. Countries such as Sweden and Finland have successfully implemented universal school food programs, and this model is increasingly appealing to policymakers in other nations aiming for similar comprehensive initiatives [38, 40, 86].

### Education

International comparative surveys such as the Program for International Student Achievement (PISA), Trends in International Mathematics and Science Study (TIMSS), and Progress in International Reading Literacy Study (PIRLS) are key for evaluating educational outcomes globally [87, 88]. Recently, these assessments have incorporated food security indicators, allowing for cross-country analyses that highlight New Zealand's high rates of food insecurity among high-income nations. Data show that students experiencing any level of household food insecurity perform substantially lower, with achievement gaps equivalent to two to four years of learning behind their food-secure peers by age 15 [37, 79].

Extensive international research confirms a strong link between household food insecurity and lower educational achievement from early childhood through adolescence, even after adjusting for confounding variables [1, 2, 89, 90]. Notably, sustained access to free school meals has been associated with improved academic outcomes, with long-term exposure (five years or more) needed to observe these benefits [38, 40, 42, 91]. Studies with long time horizons also report broader impacts, including reduced dropout rates, increased lifetime income, more productive years, and substantial public health cost savings [16, 21, 38, 40–42, 91].

Ka Ora, Ka Ako, established in 2020, shows early evidence of enhancing educational engagement. Ministry of Education surveys indicated that 64% of schools reported increased attendance following the program's implementation. Although this increase is based on teacher perceptions, qualitative evidence supports these findings [92]. A supplemental program evaluation revealed improved attendance among the most underserved students, reflecting a few additional days of school attendance over the year [92].

### Sustainability

Much more could be done within Ka Ora, Ka Ako to improve environmental sustainability outcomes. Other healthy school food programs in Brazil, Italy, Sweden, Finland, the UK and Canada incorporate waste management standards, sustainable menu planning, environmental sustainability education, procurement policies, and support for sustainable practices among local growers and suppliers in an effort to reduce greenhouse gas emissions [38, 39, 86, 93–96]. Examples of strategies being used to strengthen the sustainability credentials of school food programs include using models to balance menu taste, nutrition, and carbon footprint [94, 97], reducing barriers to sustainable food procurement [98], and improving monitoring systems [93, 95]. The power of school food programs to enhance food system sustainability and resilience is being recognized [17, 99, 100], and, in terms of the Global Syndemic of obesity, undernutrition and climate change, school food programs are considered a triple-duty action by addressing all three dimensions [17].

### Future research

Future research should prioritize the monitoring and evaluation of the new low-cost Ka Ora, Ka Ako delivery model. This is particularly important in light of multiple reports highlighting negative outcomes associated with the model’s implementation, including reduced lunch quality [101–103], diminished nutritional value [104], increased food waste [102], and greater administrative burden on schools and staff [101, 102]. Continued comprehensive data collection by the Ministry of Education will be essential to enable direct comparisons between the original and revised funding models. Additionally, a full VfI analysis of the new model should be conducted to support evidence-based decision-making and help the government determine the most effective funding approach for the program.

Despite its methodological drawbacks, a formal cost–benefit analysis for Ka Ora, Ka Ako may allow it to be directly compared with other areas of government investment. Quantitative system dynamics modelling would help to identify optimal program designs. Such models allow in-silico changes in input variables to estimate changes in outputs of interest. For example, this approach could compare the costs and impacts of the original model versus the low-cost version.

### Strengths and limitations

The participatory, comprehensive VfI system was a key strength of this study, as it empowered stakeholders—designers, funders, and recipients of Ka Ora Ka Ako—to define the program’s expected values, rate how well these values were delivered, and identify opportunities to create additional value [53, 59–61]. While the values proposed by the stakeholders predominantly focused on program effectiveness, the VfI method required a holistic evaluation, incorporating other economic domains such as economy, efficiency, equity, and cost-effectiveness, using the 5-E framework.

A wide range of evidence supported the evaluation, including both quantitative and qualitative data from local and international sources. Overall, stakeholder ratings demonstrated strong agreement, though a few criteria—such as cost-effectiveness, sustainability, and continuity—had a broader distribution of scores. This variability was largely explainable, and the active engagement of all participants throughout the process strengthened the credibility of the findings.

A few limitations are noted. One limitation is the insufficiency of quantitative data on Ka Ora, Ka Ako’s food waste and packaging waste. This is because the Ministry of Education started monitoring these aspects in 2024, so there was no prior quantification of the partly-eaten lunches or the packaging waste. Another limitation was the lack of preexisting cost-effectiveness modelling for the program. Additionally, the stakeholder rating method used in this evaluation did not include an option such as “unknown/not enough information to rate” for cases where evidence was limited. This limitation became evident during the reporting phase, particularly in relation to cost-effectiveness, where, as previously noted, national data were scarce. To address the lack of cost-effectiveness data, evaluators incorporated relevant international evidence from comparable programs in similar contexts to support stakeholder assessments. However, the absence of a “not enough information” rating may have constrained the expression of uncertainty in some areas. Future VfI evaluations of Ka Ora, Ka Ako and similar programs should consider including this option to enable more nuanced stakeholder input and better reflect areas where evidence is limited.

Additionally, while some of the study participants who defined and rated program values for the VfI analysis were policy makers, they were not decision-makers for the budget allocated to the program. Decision-makers in charge of budget allocation (i.e., ministers) may have different values or weighting of values than the participants in this study. Therefore, national budget decisions may reflect different priorities than those expressed in this study.

## Conclusions

This VfI evaluation has given a robust analysis of the value derived from the investment in Ka Ora, Ka Ako. Based on the ratings for individual criteria, we assessed Ka Ora, Ka Ako as providing very good value for investment overall. This finding is supported by the traceable evaluation logic provided by the criteria, standards and evidence used in the assessment. The results, which are comprehensive, nuanced and grounded, show that the model of Ka Ora, Ka Ako implemented between years 2019 and 2024 was performing very well against the original objectives and has the potential to create value beyond initial expectations. Free school lunch programs have such wide impacts, many of which cannot plausibly be monetized or brought into a single metric, making the VfI system a suitable methodology for considered policymaking. This evidence will inform future funding decisions by evaluating how the original program design compares, as an investment, to the new low-budget model being trialed in New Zealand from 2025. Additionally, it can contribute to the design and funding decision-making of school food interventions in other countries with similar contexts.

## Supplementary Information

Supplementary Material 1.

Supplementary Material 2.

## Data Availability

Part of the data that supported the findings of this study comes from government internal documentation available from the New Zealand Ministry of Education, but restrictions apply to the availability of these data, which were used under formal request for the current study and so were not publicly available. Such data, as well as the rest of the data used for the study, are, however, available from the corresponding author upon reasonable request and with permission from the New Zealand Ministry of Education.

## Abbreviations

NCDs: Noncommunicable diseases
PISA: International comparative surveys such as the Program for International Student Achievement
PIRLS: Progress in International Reading Literacy Study
TIMSS: Trends in International Mathematics and Science Study
VfI: Value for Investment
WHO: World Health Organization
