# Supplementary material for: Free, healthy school lunches in New Zealand: A Value for Investment analysis
Source: BMC Public Health. 2025 Oct 21;25:3546. doi: 10.1186/s12889-025-24529-8 (PMC12539167; doi:10.1186/s12889-025-24529-8)
Supplement: Supplementary file 1 — Supplementary Material 1. [file 12889_2025_24529_MOESM1_ESM.pdf]

PUBLIC HEALTH COMMUNICATION CENTRE AOTEAROA

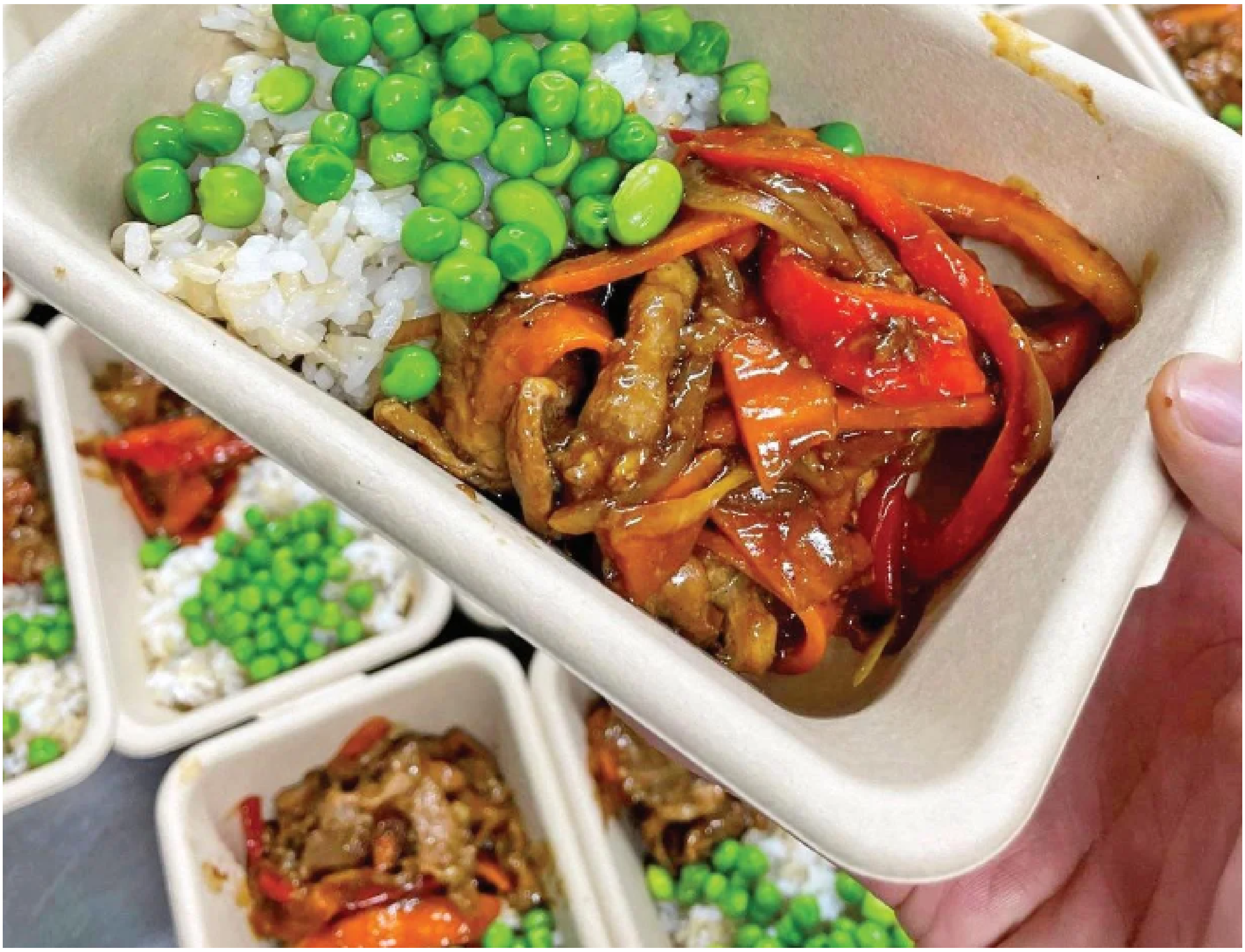

Evidence for free school lunches: Are they worth investing in?

25 March 2024

Carolina Mejia Toro, Boyd Swinburn

Summary

This Briefing considers preliminary evaluation work on Ka Ora, Ka Ako, the free, healthy school lunch programme. It finds that this programme is currently performing very well against 21 stakeholder-determined criteria. The major improvements identified are getting secure, ongoing funding for the programme; identifying ways to expand the programme so that all children in Aotearoa New Zealand will benefit; undertaking a formal cost-effectiveness study; and building in more environmental sustainability.

Value for Investment is a mixed-methods, participatory system that assesses how well resources are used and how much value is created.[1] We gathered inputs from community and government stakeholders to identify what value they expect the programme to produce. From this value proposition, we created 21 criteria in five economic domains to measure those values and validated them with stakeholders. Next, we collected Ka Ora, Ka Ako evaluations and monitoring data to create an evidence base for each criterion. Programme performance (excellent, good, adequate, poor) for each criterion was made by the research team but has yet to be validated by stakeholders. We present these preliminary assessments with more details available in the attached summary report and from the authors by request.

Evaluation findings

The summary table of evaluation criteria and provisional assessments is shown. The effectiveness criteria (in red) are the primary outcomes, based on the original Cabinet papers for the programme. [2,3]

Preliminary assessments of Value for Investment for Ka Ora, Ka Ako

DOMAINS	CRITERIA (primary outcomes in red)	ASSESSMENT
EFFECTIVENESS	Alleviating hunger at schools	EXCELLENT
	Healthy eating (lunches are healthy, safe, and high quality; promote healthy eating habits and food culture)	EXCELLENT
	Improved diet-related outcomes (mental health, healthy weight, dental health)	GOOD
	Reduced financial burden on disadvantaged households	EXCELLENT
	Strengthened local economies (local employment at living wage, local and Iwi-centred procurement and distribution)	EXCELLENT
	Reduced barriers to education and improved long-term educational outcomes (attendance, classroom engagement, educational attainment, high-school retention, curriculum links including mātauranga Māori, sustainability and nutrition)	GOOD
	Improved mana and self-esteem	EXCELLENT
	Improved community cohesion (whānau engagement with schools, improved cultural identity through food)	GOOD
	Increased food system resilience (% local food in lunches, strong and short supply lines, access to healthy affordable foods)	ADEQUATE
	Lunch menus, packaging and operations are sustainable	ADEQUATE
ECONOMY	Resources are well managed through procurement/provision/distribution policies and practices	EXCELLENT
	Fair balance of cost to govt vs quality of lunches, pay for staff and profit for providers	EXCELLENT
EFFICIENCY	Certainty of continuity of the programme	POOR
	Productive delivery (delivering healthy, safe, sufficient, locally-based meals, on time, within budget)	EXCELLENT
	Optimal level of food surplus and minimal level of food and packaging waste	EXCELLENT
	Efficiencies in design and continuous quality improvement systems in place	EXCELLENT
	Systems in place to prioritise local sustainable procurement and meal planning	GOOD
EQUITY	Across schools: the programme resources and targets schools to reach the students most in need	ADEQUATE
	Within schools: design minimises food poverty stigma	EXCELLENT
	Provisions for tailoring to school needs with policies and support systems for smaller schools and small-scale suppliers	EXCELLENT
COST-EFFECTIVENESS	The programme creates more value than it consumes based on break-even analysis of monetisable investments and benefits, and qualitative consideration of intangibles	ADEQUATE

1. Effectiveness (Is it achieving the desired outcomes/values?)

Ka Ora, Ka Ako met or exceeded expectations for its primary outcomes. Students who previously had insufficient food, reported on average feeling 20% fuller after lunch than

before the programme.[4] Furthermore, lunches meet Ministry of Health nutritional guidelines and provide more than 33% of daily nutrient requirements for growth and cognitive development.[5] Diet-related health outcomes include substantial mental health improvements (eg, 9-20% gains for children living in food-insecure homes).[6] The annual savings for households ranges from $1,000 (1 child in primary school) to $5000 (3 children in secondary school) and qualitative evidence describes reduced whānau hardship, food insecurity and time burden.[7] Local economies benefitted with about 2,500 new jobs created.[8]

Qualitative studies reported reduced barriers to education and most schools (46%-86%) reported that student attendance, engagement, behaviour, and achievement improved after the introduction of the programme.[9] A newly-published report noted improved attendance for underserved children in some school terms amounting to about 3 days/year.[10] Several New Zealand cross-sectional studies showed that food insecurity was associated with large impacts on test scores, equivalent to losing 2-4 years of schooling.[11,12]

2. Economy (Is it buying inputs of appropriate quality at the right price?)

The evidence on quality measures for the management of resources (procurement, provision, distribution) shows good adherence with all relevant government and internal Ministry policies. A fair balance is struck between costs to government versus the quality of lunches, pay for staff, and profit for providers. Staff are paid a living wage[13] and there is regular calibration of prices paid to suppliers[14] and flexibility for small and remote suppliers.[15] Complaints about food appeal and quality are rare (88 and 201 respectively in 2.5 years from approximately 1 million lunches/week).[15]

3. Efficiency (How well are inputs converted into outputs?)

The main barrier to improved efficiency is a lack of certainty about the programme's continuation. Multiple procedures and policies are in place to ensure food safety, acceptability, age-appropriateness, and cultural appropriateness.[16] The universality of lunch provision within schools contributes significantly to programme efficiency. Food surplus (untouched lunches) has improved and is now within public service standards (5-10%).[17] Almost all surplus lunches go to students or families in need or are otherwise used. There is no reliable data on food waste (partially eaten lunches) or packaging waste, but this is now built into monitoring systems.

4. Equity (How fairly are benefits distributed?)

The schools in the programme have students who are the 25% most disadvantaged, according to the Equity Index. This is fair given the total budget, but it means that the programme does not reach about 60% of students from food insecure households.[18] The universality of lunch provision within a school is strongly supported by principals and whānau to minimise food poverty stigma.[7]

5. Cost-effectiveness (How much impact does the programme have relative to the inputs invested in it?)

A formal cost-effectiveness analysis has not been done but a Canadian study estimated that free school lunches return 2.5-7-fold on investment. The 20% increase in mental health

scores among the students with food insecurity in Ka Ora, Ka Ako is a substantial intangible benefit.

Conclusions

In evaluation work to date, Ka Ora, Ka Ako is currently performing very well against 21 stakeholder-determined criteria. The major improvements identified are getting secure, ongoing funding for the programme, identifying ways to expand the programme so that all children in Aotearoa New Zealand will benefit, undertaking a formal cost-effectiveness study, and building in more environmental sustainability.

What's new in this Briefing

- We present preliminary evidence from the first Value for Investment analysis of Ka Ora, Ka Ako, the free, healthy school lunch programme. This evidence sheds light as to how well resources have been used, what kind of value has been created and what value could further be created from the resources invested.
- The evidence gathered and analysed against 21 criteria participatively built with stakeholders shows that Ka Ora, Ka Ako is performing very well, attaining its original purpose and creating further value at multiple levels: children, whānau, schools, communities, and food systems.

Implications for public policy

- Based on the current evidence, Ka Ora, Ka Ako is very good value for investment and its present reach to 25% of school children should not be cut.
- Given the wide-reaching benefits that Ka Ora, Ka Ako generates, policy options for increasing the reach of the programme should be investigated.

Authors details

Prof Boyd Swinburn, Professor of Population Nutrition and Global Health, University of Auckland and Research Professor, EIT/Te Pukenga
Carolina Mejia Toro, Doctoral candidate, Population Nutrition and Global Health, University of Auckland

The authors are involved in the Value for Investment project funded by the National Science Challenge. Professor Boyd Swinburn is also co-chair of Health Coalition Aotearoa.

This is the third in a three-part series. Read Evidence for free school lunches: The impact of hunger and learning and Evidence for free school lunches: Bigger picture benefits.

References

1. Julian King & Associates. Value for Investment. Available at: https://www.julianking.co.nz/vfi/#:~:text=The%20award%2Dwinning%20Value%20for,more%20value%20can%20be%20created. (Accessed 20 March 2024)
2. Ministry of Education. Social Wellbeing Committee. Cabinet paper - Implementing a Free and Healthy School Lunch Prototype for Year 1-8 Students. 26 June 2019. Available at https://assets.education.govt.nz/public/Uploads/R-151-153-Redacted2.pdf (Accessed 20 March 2024)
3. Ministry of Education. Cabinet Paper: Continuing the Ka Ora, Ka Ako | Healthy School Lunches Programme. 22 March 2021. Available at: https://www.education.govt.nz/assets/Documents/our-work/information-releases/Advice-Seen-by-our-Ministers/March-2021/Cabinet-Paper-material_Redacted.pdf (Accessed 20 March 2024)
4. Ministry of Education. Ka Ora Ka Ako | Healthy School Lunches: interim report. Available at: https://www.education.govt.nz/news/ka-ora-ka-ako-healthy-school-lunches-interim-evaluation-report-released/ (Accessed 20 March 2024)
5. De Seymour, Jamie, Alessandro Stollenwerk Cavallaro, Laurie Wharemate-Keung, Sheryl Ching, and Jasmin Jackson. Nutrient-Level Evaluation of Meals Provided on the Government-Funded School Lunch Program in New Zealand. Nutrients. 2022; 14(23): 5087. https://doi.org/10.3390/nu14235087.
6. Vermillion Peirce P, Jarvis-Child B, Chu L, Lennox K, Kimber N, Clarke H, et al. Ka Ora, Ka Ako New Zealand Healthy School Lunches Programme Impact Evaluation. Wellington; 2022
7. McKelvie-Sebileau P, Swinburn B, Glassey R, Tipene-Leach D, Gerritsen S. Health, wellbeing and nutritional impacts after 2 years of free school meals in New Zealand. Health Promot Int. 2023; 38(4):daad093. doi: 10.1093/heapro/daad093. PMID: 37590384; PMCID: PMC10434982
8. Aikman P, Yates-Pahulu R. He Kai Kei Aku Ringa: Evaluation of the Iwi and Hapu Social Procurement and Partnership Model, under Ka Ora, Ka Ako | The Healthy School Lunches Programme. 2023;75. Available at: KOKA He-Kai-Kei-Ringa_Evaluation-Report-FINAL-.pdf (Accessed 21 March 2024)
9. Ministry of Education. School Term Surveys for Ka Ora, Ka Ako I Healthy School Lunches Programme School Term Surveys 2021 -2023. Internal Government documentation.
10. Standard of Proof. Ka Ora, Ka Ako | New Zealand Healthy School Lunches Programme. Supplementary attendance analysis for most underserved ākonga. 13 March 2024. Available at: https://assets.education.govt.nz/public/Documents/our-work/strategies-and-policies/Ka-Ora-Ka-Ako-attendance-18-03-24.pdf (accessed 20 March 2024)
11. McKelvie-Sebileau P, Swinburn B. Food poverty for NZ teens creating an achievement lag of up to four years. 5 March 2024. Available at: https://www.phcc.org.nz/briefing/food-poverty-nz-teens-creating-achievement-lag-four-years (Accessed 19 March 2024)
12. McKelvie-Sebileau P, Railton R. Evidence for free school lunches: The impact of hunger on learning. 21 March 2024. https://www.phcc.org.nz/briefing/evidence-free-school-lunches-impact-hunger-learning Available at (Accessed 21 March 2014)
13. New Zealand Government. Procurement. Designated Contract Areas. Available at: https://www.procurement.govt.nz/broader-outcomes/designated-contract-

areas/ (Accessed 18 March 2024)
14. Ministry of Education Ka Ora, Ka Ako Healthy School Lunches. 2023 Updates for Suppliers. 9 Feb 2024. Available at: https://kaorakaako.education.govt.nz/working-together/programme-updates/2023-update-for-suppliers#december-2023 (Accessed 18 March 2024)
15. Reported through Ka Ora Ka Ako's internal complaint system (personal communication, Ministry of Education)
16. Ministry of Education. Panel Agreement Ka Ora, Ka Ako Heathy School Lunches Programme. 2023. Available at: https://assets.kaorakaako.education.govt.nz/s3fs-public/zYE1HMpokqWU3ZeDrEhsF6z2zQmehW3OZUtopRyz.pdf (Accessed 18 March 2024)
17. Gonçalves, C.; Saraiva, S.; Nunes, F.; Saraiva, C. FoodWaste in Public Food Service Sector—Surplus and Leftovers. Resources 2023; 12(120). https://doi.org/10.3390/resources12100120
18. Analysis of New Zealand PISA (Program for International Student Assessment) database (personal communication, Ministry of Education)

Public Health Expert Briefing (ISSN 2816-1203)

Source URL:
https://www.phcc.org.nz/briefing/evidence-free-school-lunches-are-they-worth-investing
