# Supplementary material for: Free, healthy school lunches in New Zealand: A Value for Investment analysis
Source: BMC Public Health. 2025 Oct 21;25:3546. doi: 10.1186/s12889-025-24529-8 (PMC12539167; doi:10.1186/s12889-025-24529-8)
Supplement: Supplementary file 2 — Supplementary Material 2. [file 12889_2025_24529_MOESM2_ESM.pdf]

Evaluating Ka Ora, Ka Ako: are school lunches worth investing in?

Value for Investment analysis of Ka Ora, Ka Ako

Boyd Swinburn, Carolina Mejia Toro, Julian King, Sally Mackay

Consent

- This workshop consists of a collective session with key experts in the Ka Ora, Ka Ako I Healthy School Lunches programme, aimed at assessing the programme's performance against a set of 21 criteria reflecting the value expected.
- The session is collective, but your individual vote is private.
- No person's rating will be visible to others or disclosed in communications and publications
- Only aggregated ratings will be shared during the session
- Process will be rapid – only 2 minutes per criterion
- Session will be recorded. Recordings will be used only for data analysis; they will not be shared with the public.

Knowing this, do you agree to participate in the ratings and be recorded?
You have the right to withdraw from the session at any time; if you withdraw, your data will not be used.

Ka Ora, Ka Ako – Value for Investment analysis

We are conducting a Value for Investment analysis of Ka Ora, Ka Ako. That is a **participatory** study to 1) identify the **value** stakeholders expect the programme to produce, 2) based on that **value proposition**, assess how value is created, for whom, how well resources are used, and **how more value can be produced** from the resources invested.

- This study is funded by the *National Sciences Challenge – A Better Start*
- Part of *Nourishing Hawke's Bay: He wairua tō te kai* project evaluating Ka Ora, Ka Ako
- Uses the award-winning Value for Investment (VfI) approach to policy and programme evaluation, developed by Dr Julian King
- Workshops (n=3) with community & government stakeholders have determined the values and criteria to assess performance
- Evidence from Ka Ora, Ka Ako evaluations, research and monitoring, national and international literature (detailed references available)

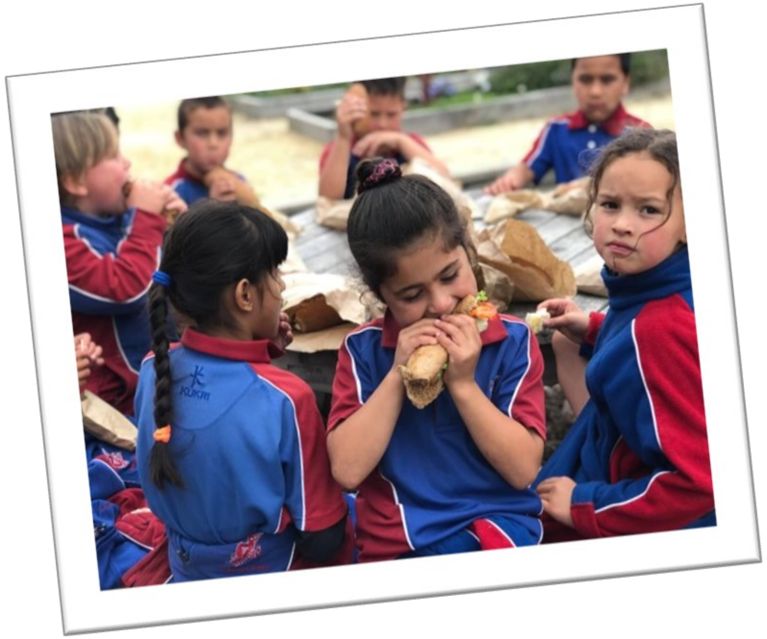

Purposes of Ka Ora, Ka Ako (based on Cabinet papers)

1. Alleviate **hunger** in schools and reduce **material hardship** in disadvantaged households

2. Improve child **nutrition** and, long-term, enhance **food choices** in adulthood and **health outcomes**

3. Increase quality, local **job** opportunities (including support for Covid economic recovery)

4. Reduce **barriers to education** (and, medium to long term, improve education outcomes)

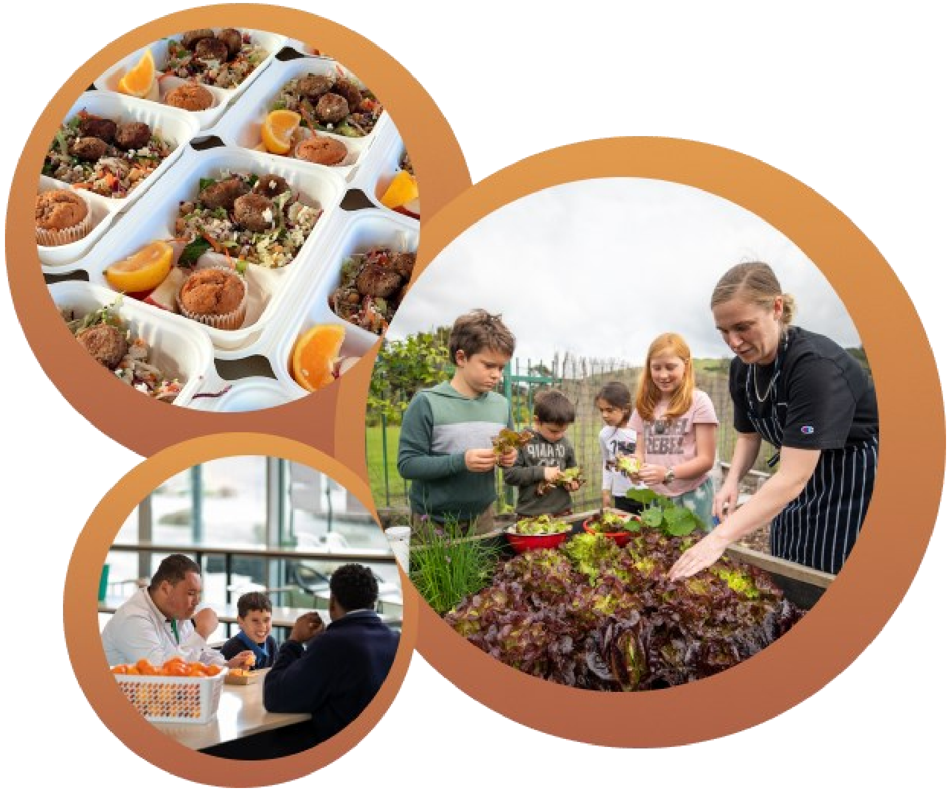

Image: https://kaorakaako.education.govt.nz/

8-step process for Value for Investment analysis

DESIGN

EVALUATION

Understand the program	Criteria	Standards	Evidence needed	Gather evidence	Analysis	Synthesis & judgement	Reporting

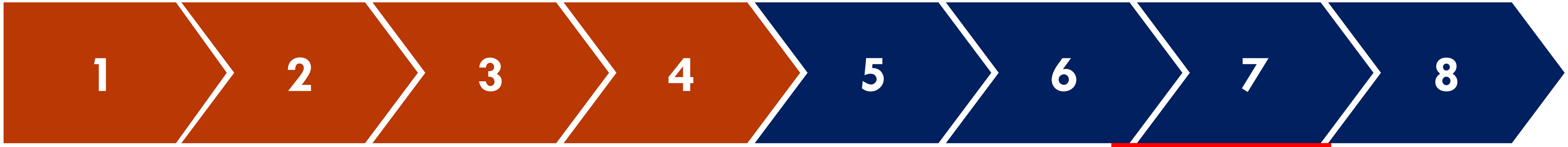

Context

What is value for the stakeholders

Definition of:

1) Criteria: aspects of good resource use and value creation

2) Standards: levels of good resource use and value creation (e.g., excellent, good, adequate and poor)

Collection of evidence to address the criteria

Analysis

Evaluative judgements using the criteria, standards and evidence

Answering:

How is value created, for whom?

How well are resources used?

Is enough value created?

How can more value be created?

© Julian King & Associates Ltd | www.julianking.co.nz

inter-disciplinary | mixed methods | evaluative reasoning | participatory

VALUE PROPOSITION Ka Ora, Ka Ako

From community stakeholders and Ministries of Education and Health

IMPROVED HEALTH AND WELLBEING

Through:
- Alleviating hunger at school
- Improving eating patterns of students and whānau
- Improving access to healthy foods
- Helping students to attain healthy growth
- Improved diet-related health outcomes (dental health, NCD risks)
- Improving mental health
- Improving mana and self-esteem
- Improving cultural identity by:
  - embedding Mātauranga Māori,
  - preventing poverty stigma,

IMPROVED LEARNING OUTCOMES

Through:
- Alleviating hunger at school and improving students' and whānau food security
- Improving students' nutritional status
- Targeting schools most in need
- Reducing the stigma of poverty (through universality design)
- Embedding manaakitanga and environmentally-sensitive nutrition education in the curriculum.
- Increasing educational attainment and classroom engagement
- Improving attendance through removing food insecurity-related barriers and improving motivation
- Increasing high-school retention

IMPROVED LOCAL ECONOMIES AND COMMUNITY COHESION

Through:
- Investing in distribution infrastructure and economically viable systems to include local food in lunches
- Enacting procurement policies to support sustainable and local content of school lunches and including Iwi procurement

IMPROVED ENVIRONMENTAL SUSTAINABILITY

Through:
- Embedding sustainability considerations in meal planning
- Embedding sustainability in procurement and contract policies
- Reducing packaging and food waste (including collaboration with food rescue organisations)
- Linking to education about sustainability
- Including more locally produced foods
- Supporting more small-scale producers

A healthy, tasty, sustainable, equitable, free school lunch system that contributes to improving food security, nutritional health and wellbeing, learning outcomes, environmental sustainability, and local economies and communities.

5-E FRAMEWORK CRITERIA FOR VALUE ASSESSMENT

1 *EFFECTIVENESS*

- Alleviating hunger at schools
- Healthy eating (lunches are healthy, safe, and high quality; promote healthy eating habits and food culture)
- Improved diet-related outcomes (mental health, healthy weight, dental health)
- Reduced financial burden on disadvantaged households
- Strengthened local economies (local employment at living wage, local and Iwi-centred procurement and distribution)
- Reduced barriers to education and improved long-term educational outcomes (attendance, classroom engagement, educational attainment, high-school retention, curriculum links including mātauranga Māori, sustainability and nutrition)
- Improved mana and self-esteem
- Improved community cohesion (whānau engagement with schools, improved cultural identity through food)
- Increased food system resilience (% local food in lunches, strong and short supply lines, access to healthy affordable foods)
- Lunch menus, packaging and operations are sustainable

2 *ECONOMY*

- Resources are well managed through procurement/provision/distribution policies and practices
- Fair balance of cost to govt vs quality of lunches, pay for staff and profit for providers

3 *EFFICIENCY*

- Certainty of continuity of the programme
- Productive delivery (delivering healthy, safe, sufficient, locally-based meals, on time, within budget)
- Optimal level of food surplus and minimal level of food and packaging waste
- Efficiencies in design and continuous quality improvement systems in place
- Systems in place to prioritise local sustainable procurement and meal planning

4 *EQUITY*

- Across schools: the programme resources and targets schools to reach the students most in need
- Within schools: design minimises food poverty stigma
- Provisions for tailoring to school needs with policies and support systems for smaller schools and small-scale suppliers

5 *COST-EFFECTIVENESS*

- The programme creates more value than it consumes based on break-even analysis of monetisable investments and benefits, and qualitative consideration of intangibles

VfI Standards for value assessment

Table 1: Generic standards (King & OPM, 2018)

Standard	Generic definition
**Excellent**	Meeting or exceeding all reasonable expectations/targets bearing in mind context. Room for incremental improvements.
**Good**	Generally meeting reasonable expectations/targets, allowing for minor exceptions. Some improvements needed.
**Adequate**	Not meeting expectations/targets but fulfilling minimum requirements and showing acceptable progress overall. Significant improvements needed.
**Poor**	Not fulfilling minimum requirements or not showing acceptable progress overall. Urgent improvements needed.

Example Question

Rate the healthiness of your breakfasts this week.

- ○ Excellent
- ○ Good
- ○ Adequate
- ○ Poor

To answer this question you will need to consider your breakfasts over the last 7 days and make a judgement using all this information.

EFFECTIVENESS: Achieving the desired outcomes/values

1. Alleviating hunger at schools

Interim evaluation found that learners who **previously had insufficient food**, on average felt **20% fuller** after lunch than they did before the programme (1-3).

Impact evaluation: **54%** of learners in **Ka Ora, Ka Ako secondary schools** had **enough food available** at school every day to feel 'just right' vs. **40%** of peer respondents who were **not recipients** of the programme (3)

(1) Nutrient-Level Evaluation of Meals Provided on the Government-Funded School Lunch Program in New Zealand
(2) New Zealand Healthy School Lunch Pilot / Ka Ora, Ka Ako Interim Evaluation, Ministry of Education
(3) Ka Ora, Ka Ako New Zealand Healthy School Lunches Programme Impact Report, Ministry of Education

EFFECTIVENESS: Achieving the desired outcomes/values

2. Improved healthy eating (lunches are healthy, safe, and high quality, promote healthy eating habits and food culture)

- Lunches are **nutritious** according to NZ reference values and international standards (1)
- Lunches meet nutrition **guidelines** and provide **>1/3 nutrient needs** for most key nutrients (1,34)
- High-quality standards and **measures in place** to ensure food safety, acceptability, age-appropriateness, and cultural appropriateness (5)
- Qualitative evidence shows improvements in **students' and whanau' eating patterns** (6,7)
- +0.9 vegetable items, -0.5 snacks items (2)
- International literature shows improved **long-term** eating behaviours (6,7)
- **Opportunities** to promote **tikanga and manaakitanga** though this could be improved in many schools (6)

(4) Healthy School Lunches Programme Nutrition Evaluation Internal Report for the Ministry of Education. 2022.
(5) Ka Ora, Ka Ako Panel Agreement
(6) Health, wellbeing and nutritional impacts after 2 years of free school meals in New Zealand. 2023
(7) Not Just a Free Lunch a logic model and evidence review for the Ka Ora, Ka Ako | Healthy School Lunch programme

EFFECTIVENESS: Achieving the desired outcomes/values

3. Improved diet-related outcomes (mental health, healthy weight, dental health)

- **Large (9-20%)** improvements in **mental health** and **wellbeing** indicators, among **food-insecure** students (2). International literature supports mental health outcomes (6,8).
- Preliminary evidence in Hawke's Bay shows that **dietary patterns** improved in Ka Ora Ka Ako schools vs non-recipient schools (analyses undergoing peer review)
- International evidence found that **exposure** to healthy school food programmes can favour the maintenance of a **healthy weight** over time and reduce the risk of **chronic diseases,** producing significant **savings in healthcare** (9,10).
- Potential to improve **dental health** (6) but not demonstrated in NZ.

(8) The Economic Rationale for Investing in School Meal Programmes
(9) Universal school meals and associations with student participation, attendance, academic performance, diet quality, food security, and body mass index: a systematic review
(10) Double- and triple-duty actions in childhood for addressing the global syndemic of obesity, undernutrition, and climate change

EFFECTIVENESS: (Achieving the desired outcomes/values)

4. Reduced financial burden on disadvantaged households

- Annual **household savings** between $1000 NZD (1 child in primary school) and $5000 (3 children in secondary school) (11)
- Qualitative findings from focus groups and interviews with whānau show **reduced hardship**, **food insecurity**, **stress**, and **time burden** (6,7)
- International literature strongly supports these findings (6, 8, 12-14).

(11) Estimations done by independent researchers using the Ka Ora, Ka Ako 2023 Price Updates for Suppliers
(12) Long-Term Effects of Childhood Nutrition: Evidence from a School Lunch Reform
(13) The Effect of Nutritious School Lunches on Education, Health, and Life-Time Income
(14) Beyond the cafeteria: The economic case for investing in school meals

EFFECTIVENESS: (Achieving the desired outcomes/values) 3/6

5. Strengthened local economies (local employment at living wage, local and Iwi-centred procurement and distribution)

- >2,455 new **jobs** created by March 2022 (>½ fulltime, all living wage) (15,16)
- **>180** business **providers**; Māori business providers supply 126 schools + iwi/hapu model supplies 40 schools (16)
- **Technical support** provided to small businesses (12)
- International literature in high-income countries shows **improvement of local economies** through generation of **local employment**, reduced socioeconomic **inequalities**, increased **productive years**, improved **opportunities** and higher **lifetime income** for children long-exposed to the programme (7,8, 12-14)

(15) Evaluation of the Iwi and Hapu Social Procurement and Partnership Model, under Ka Ora, Ka Ako | The Healthy School Lunches Programme. 2023;75. Available at: KOKA He-Kai-Kei-Ringa_Evaluation-Report-FINAL-.pdf
(16) MoE monitoring data

EFFECTIVENESS: Achieving the desired outcomes/values

6. Reduced barriers to education and improved long-term educational outcomes (attendance, classroom engagement, educational attainment, high-school retention, curriculum links including mātauranga Māori, sustainability and nutrition)

- Study (2023) revealed **reduced barriers to attendance and classroom engagement** (6)
- School term surveys reported **positive shifts** in student **attendance** (59-64%), student **engagement** (73-83%), student **behaviour** (66-74%), and student **achievement** (46-53%) (17).
- Interim evaluation showed no difference in school absenteeism (2). However, a 2024 ‘deep dive’ evaluation found an **increase of 3 days/year attendance** for underserved children (18)
- Strong impact (**2-4 years learning**) of food insecurity on NZ PISA, TIMSS, PIRLS scores (19)
- **No NZ data on education outcomes** or **high-school retention** due to the **short timeframe** of programme implementation
- International literature endorses improvement of **attendance, school engagement, educational attainment** (8, 9, 12, 13, 14), and long-term **health outcomes**, which can explain long-term improvements in **educational attainment** and lifetime **income** (12).

(17) Ministry of Education. School Term Surveys for Ka Ora, Ka Ako
(18) Supplementary analysis report for most underserved ākonga
(19) Food poverty for NZ teens creating an achievement lag of up to four years

EFFECTIVENESS: (Achieving the desired outcomes/values)

7. Improved mana and self-esteem

- Qualitative evidence for the programme being **mana-enhancing** (6, 7,15) and promoting **self-esteem** (6)
- The **universality** design prevents food poverty **stigma** (6,7)
- Improved **mental health** outcomes (9-20% in food insecure students) (2,15,20)
- International evidence supports these findings (6,7,21,22)
- Kaupapa Māori evaluation reported that:

  1. Whānau and teachers indicated that learners are better able to **enjoy** the **learning experience** and show an increased sense of **confidence** (15)

  2. The **Iwi and hapū procurement and partnership model** supports **rangatiratanga** and the **mana** of iwi and hapū in looking after their tamariki and rangatahi (15)

(20) Ka Ora, Ka Ako New Zealand Healthy School Lunches Programme Impact Evaluation
(21) Mauer S, Torheim LE, Terragni L. Children's participation in free school meals: a qualitative study among pupils, parents, and teachers.
(22) McKelvie-Sebileau P, Gerritsen S, Swinburn B, D'Souza E, Tipene-Leach D. Nourishing Hawke's Bay: He wairua tō te kai—food security, health behaviours and wellbeing in children in regional New Zealand

EFFECTIVENESS: (Achieving the desired outcomes/values)

8. Improved community cohesion (whānau engagement with schools, improved cultural identity through food)

- Qualitative evidence of increased **whānau/hapu/iwi engagement** with **schools** and **learners** through family and small-business friendly **employment**, hapu/iwi **provider models** (15,23)
- There is some evidence of engagement with **Tikanga** around kai, **socialisation** and **cultural connection** through eating together, whanaungatanga, and connections with mātauranga Māori, though these **could be strengthened** (7)
- International literature supports the potential for **increased community engagement with schools** and strengthened **community cohesion** (9, 12-14)

(23) Ka Ora, Ka Ako Approach to ordering: https://kaorakaako.education.govt.nz/working-together/guidance-and-support/approach-to-ordering

EFFECTIVENESS: (Achieving the desired outcomes/values)

9. Increased food system resilience (% local food in lunches, strong and short supply lines, access to healthy affordable foods)

- **~90%** of schools supplied by **local providers**
- However, **no data** on the impact on **community access** to healthy, affordable foods, **resilience** in emergencies
- Ka Ora, Ka Ako supports the development of **relationships** between schools/kura and iwi/hapū and **trust** between iwi and hapū and the Ministry of Education (15)
- International literature shows **potential** of healthy school meal programmes to address **climate change** (10), improve **diet sustainability** of learners and their whanau (24-26), foster **connectedness** (6,7), and improve overall **food system resilience** (26,27), including emergency preparedness and response (27,28).

(24) Oostindjer, M et al. Are school meals a viable and sustainable tool to improve the healthiness and sustainability of children´s diet and food consumption? A cross-national comparative perspective.
(25) Chaudhary A, Sudzina F, & Mikkelsen BE. Promoting healthy eating among young people—a review of the evidence of the impact of school-based interventions
(26) Hunter D, Loboguerrero AM, & Martínez Barón D. Next-generation school feeding: Nourishing our children while building climate resilience.
(27) Gupta K, Mann G, & Lambert L. P43 Conceptual Model to Assess the Resilience Capacity of School Meal Programs: A Systematic Review and Thematic Analysis
(28) Ryan Benjamin J, et al. Strengthening Food Systems Resilience Before, During and After Disasters and Other Crises.

EFFECTIVENESS: (Achieving the desired outcomes/values)

10. Lunch menus, packaging and operations are sustainable

- In 2024, **Sustainability KPIs** entered into force, requiring suppliers to implement a **waste minimisation plan** and **measure food and packaging waste to landfill** (5, 17, 23)
- To assist in the implementation of these requirements, there is **technical support** and **resources** available on the Ka Ora, Ka Ako **web** portal, designed for schools, kura and suppliers to access information, resources and guidance (29)
- No guidelines on sustainable **menu planning**
- International literature shows the power of school meals and their procurement policies in increasing **food system sustainability** (23-25)

(29) Ka Ora Ka Ako Web Portal

ECONOMY: ‘Buying inputs of appropriate quality at the right price’

11. Resources are well managed through procurement/provision/distribution policies and practices

- **Adherence** to the **Government procurement policy** ‘Achieving Broader Outcomes from Government Procurement’ (30) through the **involvement of NZ businesses** in contracts (including Māori, Pasifika, regional businesses, and social enterprises); **>8%** contracts to **Māori businesses**; ensuring at least **living wage,** and **reducing GHG emissions and waste** (5, 15, 17, 23, 29)

- Multiple **internal MoE policies** for good resource and process management with suppliers and schools (3, 5, 15, 17, 23)

(30) Achieving Broader Outcomes from Government Procurement

ECONOMY: 'Buying inputs of appropriate quality at the right price'

12. Fair balance of cost/quality to government vs quality of lunches, pay for staff and profit for providers

- Lunch quality: very **rare complaints** on menu appeal (n=88) and quality (n=201) since Nov 2021 (from ~1M lunches/week) (17)
- Pay for staff: Employment **at least at a living wage** (procurement provisions) (5, 15, 29, 30)
- **Profit for suppliers:** Each term close calibration of **pricing/lunch** to **inflation** (11, 16)
- **Flexibility:** Options for **small** and **remote providers** (5)

EFFICIENCY: ‘How well are inputs converted into outputs?’

13. Certainty of continuity of the programme

- Programme **funded year-on-year** from Covid recovery funding with no certainty of ongoing funding (31,32)
- Substantial **school and provider feedback** that lack of continuity is a **major barrier to investing** in systems, people, supply chains, and equipment for improved efficiency (16,17).
- **News** that the programme will run for at least 2 more years under a new model with a substantially lower budget (33).

(31) Ministry of Education. Social Wellbeing Committee. Cabinet paper - Implementing a Free and Healthy School Lunch Prototype for Year 1-8 Students. 26 June 2019
(32) Ministry of Education. Cabinet Paper: Continuing the Ka Ora, Ka Ako | Healthy School Lunches Programme. 22 March 2021
(33) McConnel G. Is the Government going to cut back free school lunches? Stuff. 12 April 2024

EFFICIENCY: ‘How well are inputs converted into outputs?’

14. Productive delivery of healthy, safe, sufficient, appealing, locally-based meals, on time, within budget

- Lunches meet nutrition **guidelines** and provide **>1/3 nutrient needs** for most key nutrients (1,34)
- Provision for **special diets** (16)
- **Food safety, portion sufficiency** and **acceptability** (5,16) **standards**. All suppliers must comply.
- **Rare** food **safety/suitability incident** reports (only 56 identified) (17)
- **Rare incident** reports on **late deliveries** (only 91) (17)
- **Satisfaction** surveys: **High ratings** of programme success from schools (4.5/5) and suppliers (4.7/5) (17)
- Detailed **logistics for delivery** for each supplier (5,23)
- Staying **within budget** allocations despite inflation increases in costs (11,16)

EFFICIENCY: ‘How well are inputs converted into outputs?’

15. Optimal level of food surplus and minimal level of food and packaging waste while ensuring coverage for students

- School surveys: Mean food surplus (untouched lunches) <10%; of that surplus, 58% is **distributed among students in need**, 21% goes to **food rescue**, 9% is managed by supplier, 11% other (17).
- Provider surveys: Mean food surplus <5%; of that surplus, 56% is **stored/distributed** by schools, 28% to **food rescue** organisations, 6% to landfill, 10% other (16).
- **No data on food waste** (partly eaten lunches) and **packaging waste** to landfill at the moment
- **New systems** implemented in 2024 to (1) **match more closely** the **number of lunches** delivered with number of **students expected** on the day and (2) measure **waste to landfill** and **surplus** (16)
- **All providers** have waste minimisation plans, count on **guidance materials** and technical support (5,16, 29,30)

EFFICIENCY: ‘How well are inputs converted into outputs?’

16. Efficiencies in design and continuous quality improvement systems in place

- Works within-school **universality** (provision of lunches to all students), a highly efficient design (6,36,37,38)
- **Monitoring surveys** of schools and providers each term (uptake, surplus, satisfaction)
- Systems in place to **report incidents**, file and process **complaints**
- Regular **MoE monitoring** (e.g., absenteeism, stand downs) (16,17)
- **Detailed contracts and KPIs** with performance management provisions (5,23,30)
- Regular **menu reviews** against the standards (1,16)
- Substantial **technical support** (5,15)
- Programme **evaluations** (4 completed, 1 near completion) (40); nutrition evaluation (40)
- **Per lunch payments adjusted** each term based on inflation (11,16)

(36) Andreyeva T, Sun X. Universal School Meals in the US: What Can We Learn from the Community Eligibility Provision?
(37) Leos-Urbel, Jacob, et al. "Not just for poor kids: The impact of universal free school breakfast on meal participation and student outcomes.“
(38) Ruetz, Amberley T., and Mary L. McKenna. "Characteristics of Canadian school food programs funded by provinces and territories.“
(40) Ministry of Education NZ. Evaluating Ka Ora, Ka Ako

EFFICIENCY: 'How well are inputs converted into outputs?'

17. Systems in place to prioritise local sustainable procurement and meal planning

- **Some** sustainability **provisions** in the provider contract **KPIs**: waste minimisation plans, surplus and waste monitoring (5,29)
- Sustainability **support materials** and **guidelines** on Ka Ora, Ka Ako website (29)
- Assessment of **carbon footprint** of lunches completed (16b)
- **No** assessments of **menu sustainability** (16)

(16b) Reducing emissions in schools and kura – Education in New Zealand

EQUITY: 'How fairly are benefits distributed?'

18. Across schools: the programme resources and targets schools to reach the students most in need

- Programme design uses **Equity Index** to identify the schools with students facing the **greatest socioeconomic barriers**; 25% of students in NZ attend eligible schools (29,41,42).
- **Not stopped** if a school's Equity Index rises above the 25% threshold.
- However, the **majority (~60%)** of students from households with **food insecurity** in NZ are **not covered** (43).

(41) Ministry of Education NZ. Ka Ora, Ka Ako | Healthy School Lunches Programme. How do Schools and Kura take part? – The Equity Index
(42) Ministry of Education NZ. The Equity Index.
(43) Analysis of New Zealand PISA (Program for International Student Assessment) database (personal communication, Ministry of Education)

EQUITY: 'How fairly are benefits distributed?'

19. Within schools: design minimises food poverty stigma

- **Universality** design (provision of lunches to all students within the school) minimises food poverty **stigma** and improves **uptake** (7)
- This finding is strongly supported by international evidence (36-39)

(36) Andreyeva T, Sun X. Universal School Meals in the US: What Can We Learn from the Community Eligibility Provision?
(37) Leos-Urbel, Jacob, et al. "Not just for poor kids: The impact of universal free school breakfast on meal participation and student outcomes."
(39) Ruetz, Amberley T., and Mary L. McKenna. "Characteristics of Canadian school food programs funded by provinces and territories."

EQUITY: ‘How fairly are benefits distributed?’

20. **Provisions for tailoring to context, needs and culture of schools and whānau with policies and support systems for smaller schools and small-scale suppliers**
    - Several **delivery models** available.
    - **Schools can choose** and set up their model according to context and **needs**
    - **Support systems** in place, especially for schools with challenging circumstances (e.g., remoteness, special needs) (5,15).
    - **Flexibility** for **small suppliers** and support systems, including training, sourcing technical expertise, and reducing administrative burden (15,16)
    - Very **little negative feedback** in school and provider surveys on **design** and **support** issues apart from some push-back on programme rigidity around the organisation of cultural events (e.g., Matariki, cultural days) (16,17)

COST-EFFECTIVENESS: ‘How much impact does the programme have relative to the inputs invested in it?’

21. The programme creates more value than it consumes based on break-even analysis of monetizable investments and benefits, and qualitative consideration of intangibles

- Investment of ~$325M/year (as at April 2024)
- Monetisable benefits depend on the **time horizon**
- No formal analysis for cost-effectiveness in NZ. Yet, formal analyses on the return on investment in Canada suggest a **2.5 -7x benefit** (8,14)
- In Sweden, pupils exposed to the programme during the entire primary school period had 3% **higher lifetime income** (12,13)
- Many **intangible benefits** in NZ, including a 20% increase in **mental well-being** among students with food insecurity (2,3)
- International evidence from school lunch programme evaluations shows that it can take **up to five years** before the benefits to learning and educational achievement are observed (14B).
- International evidence also shows that **return is higher than investment** through state **savings in healthcare** for chronic diseases and mental health, increased productive years of life, savings in welfare, increased **human capital** from improved **educational attainment**, reduced school **dropout**, improved **job opportunities** and **income** (8,12-14).

(14B) School feeding and learning achievement: Evidence from India’s midday meal program
